# INTRA-ABDOMINAL PRESSURE MONITORING DURING LÁZARO DA SILVA’S PROCEDURE FOR VENTRAL HERNIA REPAIR: A CROSS-SECTIONAL STUDY

**DOI:** 10.1590/0102-6720202400020e1813

**Published:** 2024-07-19

**Authors:** Pedro Ducatti de Oliveira E Silva, Renato Miranda de Melo, Cássio Eduardo da Silva Gontijo, Ênio Chaves de Oliveira

**Affiliations:** 1 Universidade Federal de Goiás, Faculty of Medicine, Postgraduate Program in Health Sciences – Goiânia (GO), Brazil; 2 Hospital Geral de Goiânia Dr. Alberto Rassi – Goiânia (GO), Brazil; 3 Universidade Federal de Goiás, Faculty of Medicine, Department of Surgery – Goiânia (GO), Brazil; 4 Hospital Universitário de Brasília – Brasília (DF), Brazil.

**Keywords:** Ventral Hernia, Incisional Hernia, Intra-Abdominal Hypertension, Herniorrhaphy, Hérnia Ventral, Hérnia Incisional, Hipertensão Intra-Abdominal, Herniorrafia

## Abstract

**BACKGROUND:**

Maintaining normal intra-abdominal pressure (IAP) levels must be one major outcome of any ventral hernia repair, avoiding hypertension or abdominal compartment syndrome.

**AIMS:**

To evaluate IAP during ventral hernia repair using Lázaro da Silva’s procedure.

**METHODS:**

IAP measurements using intravesical pressure were performed during four crucial intraoperative moments. Twenty-eight patients submitted to incisional herniorrhaphy were analyzed.

**RESULTS:**

The IAP increased by 0.5 mmHg during the procedure, regardless of the type of prior laparotomy, sex, age, obesity, or hernia width.

**CONCLUSIONS:**

Despite the IAP increase observed, Lázaro da Silva’s procedure did not result in intra-abdominal hypertension or abdominal compartment syndrome.

## RESUMO

**RACIONAL:**

A manutenção dos níveis normais de pressão intra-abdominal (PIA) deve ser um dos principais resultados de qualquer correção de hérnia ventral, evitando hipertensão ou síndrome do compartimento abdominal.

**OBJETIVOS:**

Avaliar a PIA durante a correção de hérnia ventral pela técnica de Lázaro da Silva.

**MÉTODOS:**

As medidas da PIA por meio de pressão intravesical foram realizadas durante quatro momentos cruciais do intraoperatório. Foram analisados 28 pacientes submetidos à herniorrafia incisional.

**RESULTADOS:**

A PIA aumentou 0,5 mmHg durante o procedimento, independentemente do tipo de laparotomia prévia, sexo, idade, obesidade ou extensão da hérnia.

**CONCLUSÕES:**

Apesar do aumento da PIA observado, o procedimento de Lázaro da Silva não resultou em hipertensão intra-abdominal ou síndrome do compartimento abdominal.

## INTRODUCTION

Ventral hernia (VH) is considered a frequent complication after laparotomies, occurring in 10 to 23% of cases, or even at higher frequencies, according to the analyzed sampleg^1^. It is a miscellaneous disease, with a broad severity spectrum, and yet a consensus defining a so-called "complex hernia" does not exist. Depending on the defect position on the abdominal wall, there are many surgical procedures to treat VH; the midline defects (both upper and lower) are the most frequent, also with higher surgical technique alternatives^11,13^.

Ventral hernia repair (VHR) involves some challenges, such as reconstructing the abdominal wall while returning the herniated content to the abdominal cavity without significantly raising intra-abdominal pressure (IAP). Due to its physiopathology, midline VHs promote shortening of the abdominal lateral musculature and lowering of the diaphragmatic dome, leading to a volumetric reduction of the abdominal cavity. In consequence, returning the herniated content to a smaller abdominal cavity can develop intra-abdominal hypertension (IAH), leading to abdominal compartment syndrome (ACS) and catastrophic consequences for the patient^2,13^. Therefore, achieving intra- and postoperative normal IAP levels should be an immediate outcome of VHR.

The VHR procedure developed by Alcino Lázaro da Silva (ALS), a former Brazilian surgeon, to treat large midline defects, has achieved good outcomes over the last 50 years^6,7^. This is considered a tissue repair, thus sparing the use of synthetic meshes — majorly polypropylene. The advantage of this type of VHR can be observed in the long term, as synthetic meshes present with long-term complication rates of 5% and similar recurrence rates^4,5^. Regarding this, Lazaro da Silva’s procedure shows long-term recurrence rates of 7.7%, similar to VHR employing synthetic meshes^7^.

Clinically, it is relevant to anticipate ACS and to guarantee that this complication does not occur during VHR of large defects, despite other preoperative efforts, such as preoperative pneumoperitoneum or botulinum toxin type A. Monitoring intra-abdominal pressure is a measure that can early diagnose IAH in order to avoid ACS.

Therefore, the purpose of this study was to evaluate IAP raising during Lázaro da Silva’s procedure for VHR, using intravesical pressure (IVP) monitoring.

## METHODS

**Figure 1:**
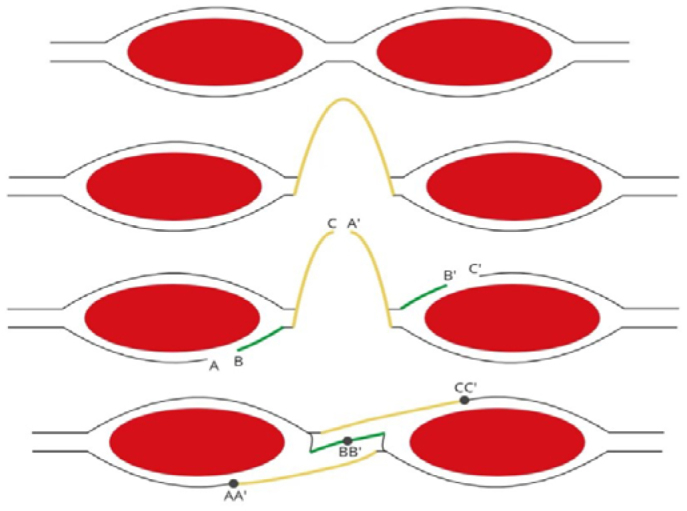
Schematic representation of a transversal section of the Lázaro da Silva’s procedure (i.e., longitudinal bilateral peritoneum-aponeurotic transposition). A: posterior-lateral flap; B: posterior-medial flap; C: right sac flap; A’: left sac flap; B’: anterior-medial flap; C’: anterior-lateral flap; AA’: first suture plane; BB’: second plane; CC’: third plane.

**Figure 2:**
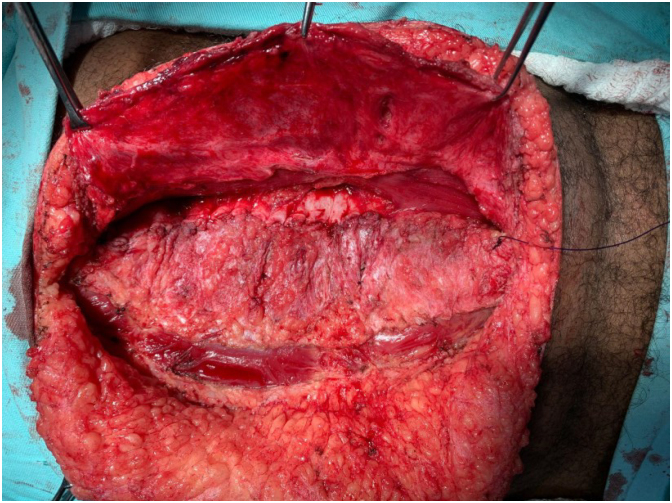
Confection of the end of the first suture plane.

**Figure 3:**
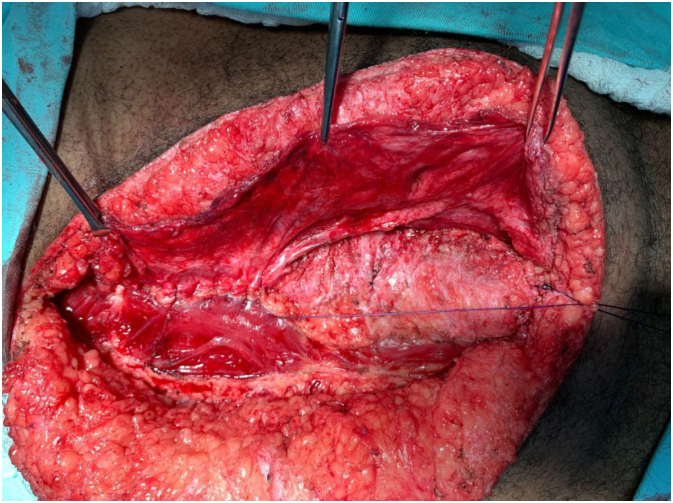
Confection of the second suture plane.

**Figure 4:**
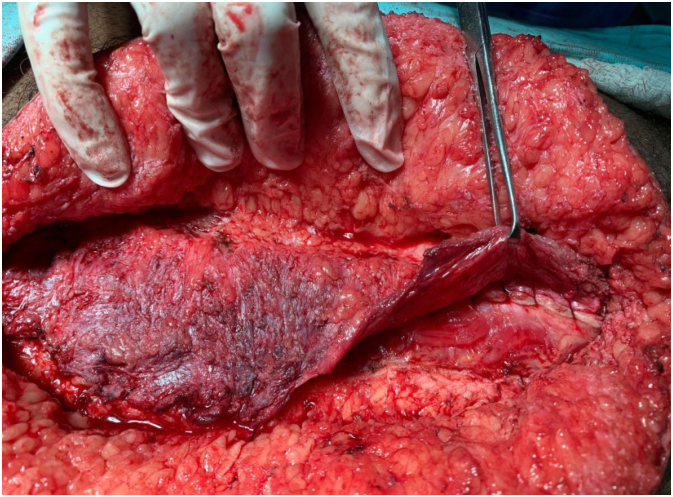
Confection of the third suture plane.

This is a cross-sectional and prospective study in which IAP was measured during VHR. Lázaro da Silva’s procedure, which is a bilateral longitudinal peritoneum-aponeurotic transposition described in 1971 (Figure 1), was chosen for the VHR. Figures 2, 3, and 4 show intraoperative photos of the Lázaro da Silva’s procedure.

Intra-operatively, IAP was measured through IVP (in mmHg) according to Kron’s method, using a 16-Fr Foley catheter and an electronic pressure transducer^9^. The IVP measurement occurred at four distinct surgical moments: immediately before surgical incision (moment 0; or t0) and after each one of the three suture planes of the VHR technique (moments 1, 2 and 3; or t1, t2, and t3). All patients had general anesthesia and epidural block.

This study included patients between 18 and 80 years old, presenting midline incisional hernias, without loss of domain, classified according to the European Hernia Society (EHS) ventral hernia classification^10^: M (midline) – M1 (subxiphoidal), M2 (epigastric), M3 (umbilical), M4 (infraumbilical), and M5 (suprapubic); W (width) – W1 (less than 4 cm), W2 (between 4 and 10 cm), and W3 (more than 10 cm). Patients were excluded when the original Lázaro da Silva’s procedure was deemed not feasible.

The following parameters were evaluated: sex, age, prior laparotomy, enterotomy, obesity (body mass index [BMI] - kg/m^2^), diabetes mellitus, and smoking.

For IVP-related data, we employed central trend, symmetry, and value dispersion through analysis of variance (ANOVA), followed by *post-hoc* analysis using Bonferroni’s correction method. Statistical Package for Social Science (SPSS), version 26 (IBM Corporation, Armonk, U*SA*) was employed for data analysis. The study was approved by the Ethics Committee of the institution (number 45878621.5.0000.0035).

## RESULTS

Initially, 30 patients were included. Two of them were excluded from the analysis because the original Lázaro da Silva’s technique was not feasible. Demographic data of the remaining 28 patients are reported in Table 1. The sample was mostly female (64.3%), with conventional gastroplasty as prior laparotomy in 53.6%, and hernia diameter defect between 4 and 10 cm (EHS-W2) in 67.9%; 50.0% of the patients were over 60 years old, and 50.0% were obese (BMI>30 kg/m^2^).

**Table 1:** Demographic and anthropometric characteristics of the patients.

		n=28
		Mean±SD
Age (years)		56.32 ± 13.55
BMI (kg/m^2^)		28.84 ± 5.32
Defect length (cm)		18.89 ± 5.20
Defect width (cm)		9.25 ± 3.43
		n (%)
Age group		
	<60 years	14 (50.0)
	≥60 years	14 (50.0)
Sex		
	Female	18 (64.3)
	Male	10 (35.7)
Prior laparotomy		
	Gastroplasty	15 (53.6)
	Others	13 (46.4)
Obesity		
	No	14 (50.0)
	Yes	14 (50.0)
Diabetes mellitus		
	No	23 (82.1)
	Yes	5 (17.9)
Smoking		
	No	25 (89.3)
	Yes	3 (10.7)
Enterotomy		
	No	23 (82.1)
	Yes	5 (17.9)
EHS-M		
	M1–M2	4 (14.3)
	M1–M3	15 (53.6)
	M1–M4	3 (10.7)
	M1–M5	2 (7.1)
	M2–M3	1 (3.6)
	M2–M4	1 (3.6)
	M2–M5	1 (3.6)
	M3–M5	1 (3.6)
EHS-W		
	W2	19 (67.9)
	W3	9 (32.1)

n: absolute frequency; %: relative frequency; SD: standard deviation; BMI: body mass index; M: midline; W: width; EHS-M: European Hernia Society midline; EHS-W: European Hernia Society width.

**Figure 5:**
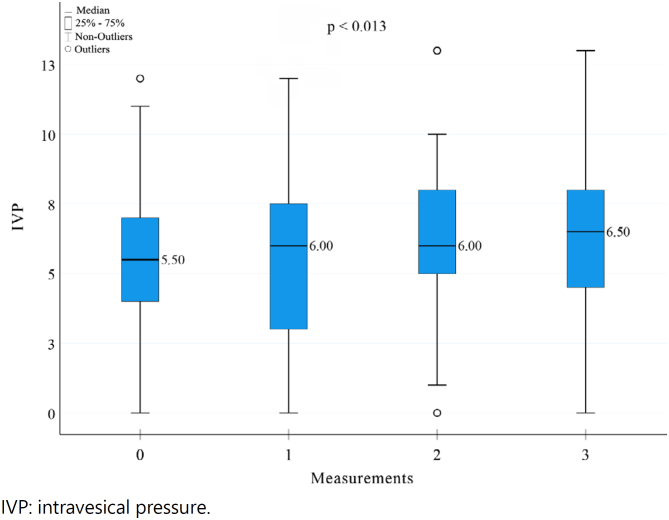
Boxplot graphic with intravesical pressure results on the moments: before skin incision, and after each one of the suture planes of Lázaro da Silva’s procedure, moments 0, 1, 2 and 3 respectively.

Mean values of IVP measured intra-operatively were: t0=5.53 (standard deviation [SD]±2.83) mmHg; t1=5.61 (±3.03) mmHg; t2=6.22 (±3.28) mmHg; and t3=6.19 (±2.27) mmHg. Median values were 5.50 mmHg, 6.00 mmHg, 6.00 mmHg, and 6.50 mmHg, respectively. There was a significant difference between the samples (p-value [p]<0.010). To identify the moment this difference occurred, a *post-hoc* analysis comparing each of the moments was performed (Figure 5). As a result, there was a significant difference only between moments t0 and t2 (p=0.019, p<0.050), with a 0.50 mmHg IAP raising. However, IAP values did not exceed normal pressure (i.e., lower than 12 mmHg).

In subgroups *post-hoc* analysis, interaction between IVP values of the following subgroups was tested: prior laparotomy (gastroplasty *vs.* other procedures; p=0.984); sex (male *vs.* female; p=0.981); age (<60 years *vs.* ≥60 years; p=0.971); obesity (BMI<30 kg/m^2^ *vs.* BMI≥30 kg/m^2^; p=0.967); and defect width (EHS-W2 *vs.* EHS-W3; p=0.981). In summary, there was no significant difference in all studied groups.

## DISCUSSION

The results showed that Lázaro da Silva’s procedure can reconstruct the abdominal wall without raising the IAP. Variation in the physiologic values of IAP could have occurred and definitely happened. However, this elevation was anticipated, as the second suture plane (t2) involved suturing the aponeurotic defect and reconstructing the linea alba, while the other planes (t1 and t3) acted as lateral anchoring, which interfered less with the IAP values. Post-gastroplasty patients represented half of the study sample, which was initially a concern. However, subgroup analysis of prior laparotomy showed no significant difference (p=0.984, p>0.050).

Furthermore, VHR of an EHS-W2 without-loss-of-domain hernia (i.e., width between 4 and 10 cm) could rarely promote IAH or ACS, so this subgroup analysis was also relevant. Similarly to the other groups, there were no significant differences between EHS-W2 and EHS-W3 hernias. Plausible mechanisms for this phenomenon could be Lázaro da Silva’s procedure itself: the relaxing incisions and the employment of the hernial sac could enlarge the abdominal cavity and raise its compliance.

Our data corroborate another experiment, using the same surgical procedure. In that experiment, ALS himself measured the IAP in 31 patients with a Teflon transabdominal catheter and registered similar values pre- and post-operatively^8^. Unlike in our study, IAP measures were not gathered intraoperatively nor were the anesthesia characteristics described (i.e., type of anesthesia, ventilation control, and neuromuscular blockade). Despite that, employing a controlled method and observing still intraoperatively, our study re-evaluated and confirmed the results obtained from that pioneer experiment.

In concern with ACS, Kirkpatrick et al. described its occurrence in VHR, developing the concept of quaternary abdominal compartment syndrome^3^. In illustration, working with the worst cases, another study evaluating 115 hernia patients with loss of domain showed 92% of IAH and 16% of ACS after VHR^12^, using techniques other than Lázaro da Silva’s procedure. In our sample, which had no loss of domain, we did not observe any clue of IAH. However, strict intraoperative checking of IAP is a fundamental measure in treating more complex hernias, as Quintela et al. observed higher rates of HIA and SCA^12^.

Limitations of this study can be summarized in sample characterization, despite *post-hoc* analysis not demonstrating any impact on results. Evaluating and measuring IAP during Lázaro da Silva’s procedure in the worst scenarios, like larger defects or with loss of domain, will be central to confirm the results obtained in this study. At least, in future research, we suggest a larger sample in order to encompass different clinical scenarios, though we recognize the difficulty in routinely measuring IAP.

## CONCLUSIONS

Lazaro da Silva’s procedure is a safe VHR technique and does not raise IAP. Variations in IAP can occur within its physiological values and without causing clinical issues. Also, subgroup analysis of age, sex, prior laparotomy, diabetes mellitus, smoking, hernia width, and obesity did not interfere with IAP in this study.
